# DXP Synthase Function in a Bacterial Metabolic Adaptation and Implications for Antibacterial Strategies

**DOI:** 10.3390/antibiotics12040692

**Published:** 2023-04-01

**Authors:** Eric C. Chen, Caren L. Freel Meyers

**Affiliations:** Department of Pharmacology and Molecular Sciences, The Johns Hopkins School of Medicine, Baltimore, MD 21201, USA

**Keywords:** 1-deoxy-d-xylulose 5-phosphate synthase, DXPS, MEP, DsdA, d-serine, bacterial adaptation, urinary tract infection, CoA inhibition, acetylphosphonate, cofactor inhibition

## Abstract

Pathogenic bacteria possess a remarkable ability to adapt to fluctuating host environments and cause infection. Disturbing bacterial central metabolism through inhibition of 1-deoxy-d-xylulose 5-phosphate synthase (DXPS) has the potential to hinder bacterial adaptation, representing a new antibacterial strategy. DXPS functions at a critical metabolic branchpoint to produce the metabolite DXP, a precursor to pyridoxal-5-phosphate (PLP), thiamin diphosphate (ThDP) and isoprenoids presumed essential for metabolic adaptation in nutrient-limited host environments. However, specific roles of DXPS in bacterial adaptations that rely on vitamins or isoprenoids have not been studied. Here we investigate DXPS function in an adaptation of uropathogenic *E. coli* (UPEC) to d-serine (d-Ser), a bacteriostatic host metabolite that is present at high concentrations in the urinary tract. UPEC adapt to d-Ser by producing a PLP-dependent deaminase, DsdA, that converts d-Ser to pyruvate, pointing to a role for DXPS-dependent PLP synthesis in this adaptation. Using a DXPS-selective probe, butyl acetylphosphonate (BAP), and leveraging the toxic effects of d-Ser, we reveal a link between DXPS activity and d-Ser catabolism. We find that UPEC are sensitized to d-Ser and produce sustained higher levels of DsdA to catabolize d-Ser in the presence of BAP. In addition, BAP activity in the presence of d-Ser is suppressed by β-alanine, the product of aspartate decarboxylase PanD targeted by d-Ser. This BAP-dependent sensitivity to d-Ser marks a metabolic vulnerability that can be exploited to design combination therapies. As a starting point, we show that combining inhibitors of DXPS and CoA biosynthesis displays synergy against UPEC grown in urine where there is increased dependence on the TCA cycle and gluconeogenesis from amino acids. Thus, this study provides the first evidence for a DXPS-dependent metabolic adaptation in a bacterial pathogen and demonstrates how this might be leveraged for development of antibacterial strategies against clinically relevant pathogens.

## 1. Introduction

Central metabolism is an attractive target space for antibacterial development as the metabolic state of pathogenic bacteria is closely linked to survival and pathogenicity, as well as susceptibility to antibiotics [1,2,3]. The host environment plays a role in this metabolic state as bacteria must rapidly remodel their metabolism to survive, colonize and cause infection within a specific host environment [4,5] and in the face of a mounting immune response. Thus, impairing bacterial metabolic processes that are essential under specific conditions within the host and/or for adaptation to fluctuating host environments is a promising approach to prevent or treat infection [6,7,8,9,10,11,12]. 

**Figure 1:**
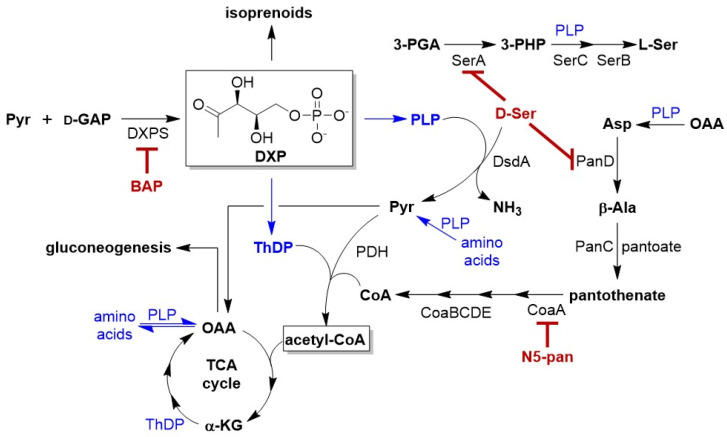
Proposed role of DXPS in UPEC adaptation to d-Ser. Potential metabolic vulnerabilities caused by inhibiting DXPS in the context of UTI include increased sensitivity of UPEC to inhibition of l-serine and/or CoA biosynthesis (red). Metabolic steps involved the TCA cycle or in l-serine/CoA biosynthesis that require PLP or ThDP are highlighted in blue. Abbreviations: acetyl-CoA (acetyl coenzyme A), Asp (aspartate), PanD (aspartate decarboxylase), β-Ala (β-alanine), CoA (coenzyme A), DXP (1-deoxy-d-xylulose 5-phosphate), DXPS (1-deoxy-d-xylulose 5-phosphate synthase), CoaE (dephospho-CoA kinase), d-GAP (d-glyceraldehyde 3-phosphate), α-KG (α-ketoglutarate), OAA (oxaloacetate), PanC (pantothenate synthase), CoaA (pantothenate kinase), 3-PGA (3-phosphoglycerate), SerA (3-phosphoglycerate dehydrogenase), 3-PHP (3-phosphohydroxypyruvate), CoaD (4′-phosphopantetheine adenylyltransferase), CoaB (4′-phosphopantothenoylcysteine synthetase), CoaC (4′-phosphopantothenoylcysteine decarboxylase), SerC (3-phosphoserine aminotransferase), SerB (phosphoserine phosphatase), PLP (pyridoxal 5-phosphate), Pyr (pyruvate), PDH (pyruvate dehydrogenase), d-Ser (d-serine), DsdA (d-serine deaminase), l-Ser (l-serine), ThDP (thiamin diphosphate).

A promising drug target to develop in this context is the essential bacteria-specific metabolic enzyme, 1-deoxy-d-xylulose 5-phosphate synthase (DXPS). DXPS catalyzes the formation of DXP [13,14,15,16] which sits at a metabolic branchpoint (Figure 1), serving as a precursor to isoprenoids as well as the vitamins thiamin diphosphate (ThDP) and pyridoxal-5-phosphate (PLP). Given the importance of these metabolites in a myriad of central metabolic processes, we have hypothesized that DXPS plays a critical role in bacterial adaptation and survival in fluctuating host environments during infection, and thus is a potential drug target [17,18,19,20,21]. However, to our knowledge, DXPS functions in specific pathogen adaptations have not yet been reported. 

DXPS-catalyzed formation of DXP from pyruvate and d-glyceraldehyde 3-phosphate (d-GAP) requires the cofactor ThDP for chemical conversions that are reminiscent of other ThDP-dependent pyruvate decarboxylase and carboligase enzymes [20]. However, previous work from our lab has shown that DXPS possesses unique structural features and exhibits a distinctive enzymatic mechanism, which has enabled the development of selective inhibitors. Alkyl acetylphosphonates (alkylAPs), including butyl acetylphosphonate (BAP), were developed to selectively target the large DXPS active site. These substrate analogs inhibit bacterial cell growth in a medium-dependent manner through a mechanism involving inhibition of DXPS [17,18,19], and thus can be used as cellular probes of DXPS function and potential starting points for DXPS-targeted antibiotic development. 

Toward establishing its function in pathogen adaptation and its potential as an antibacterial drug target, we are investigating DXPS inhibition in the context of urinary tract infection (UTI) which is among the most common of bacterial infections worldwide [22,23]. Antibiotics are used extensively to treat UTIs, which in turn promotes drug-resistant infections that can progress to life-threatening urosepsis. The majority of UTIs are caused by uropathogenic *E. coli* (UPEC) originating from the gut, a vastly different environment than the urinary tract. UPEC colonize and ascend the urinary tract, shifting metabolism to adapt to nutrient-diverse milieus of the urine, bladder/kidney and ultimately the bloodstream. In the urine, small peptides and amino acids are a main carbon source for UPEC [4,5,24] metabolized to TCA cycle intermediates, providing key precursors for gluconeogenesis, amino acid biosynthesis, energy metabolism, etc. [24,25,26,27]. DXPS-dependent vitamin biosynthesis is expected to be critical for UPEC adaptation to this environment within the urinary tract.

d-Serine (d-Ser) is a particularly important metabolite in urine [4,28,29], present at concentrations up to 1 mM, approximately 1000-fold higher than its concentration in the gut [30]. d-Serine is also toxic to bacteria, exerting bacteriostatic effects through inhibition of l-serine and pantothenate biosynthesis [31,32,33]. However, most UPEC clinical isolates harbor the *dsdCXA* tolerance locus whose gene products are responsible for transport and catabolism of d-Ser [29,33,34,35,36,37,38], enabling UPEC to grow in its presence. The *dsdA* gene encodes the enzyme d-serine deaminase (DsdA) which catalyzes the PLP-dependent conversion of d-Ser to pyruvate, thus serving a role in detoxification of d-Ser and allowing for its utilization as a nitrogen source and precursor to gluconeogenesis [39,40]. We hypothesize that DXPS plays a role in pathogen adaptation to d-Ser. Inhibition of DXPS-dependent PLP synthesis should limit the PLP-dependent catabolism of d-Ser by DsdA, leading to its accumulation and bacteriostatic effects through inhibition of l-serine and pantothenate biosynthesis (Figure 1). 

Here we have investigated the role of DXPS in UPEC adaptation and provide the first evidence that perturbing bacterial central metabolism through DXPS inhibition not only impairs bacterial growth but also hinders the ability of bacteria to adapt to new environments. Specifically, our results suggest that inhibiting DXPS impairs a UPEC adaptation within the urinary tract by preventing detoxification of d-Ser and its utilization for gluconeogenesis. Additionally, we show that DXPS inhibition creates a metabolic vulnerability that sensitizes UPEC to inhibition of CoA and/or l-Ser biosynthesis. Together, vitamin and CoA biosynthesis are required for a functioning TCA cycle and gluconeogenesis, and thus critical for UPEC survival in the urinary tract. Thus, our work suggests a potential Achilles’ heel in UPEC metabolism; combining inhibitors of DXPS and CoA biosynthesis (Figure 1) could serve as an effective strategy to impair adaptation and growth of UPEC in the context of UTI. The results from this study illustrate DXPS as an important target for the development of antibacterial strategies that exploit bacterial metabolic adaptations in host environmental niches, enabling the potential development of more targeted therapies. 

## 2. Results

### 2.1. Impairing DXPS Increases UPEC Sensitivity to d-Ser

**Figure 2:**
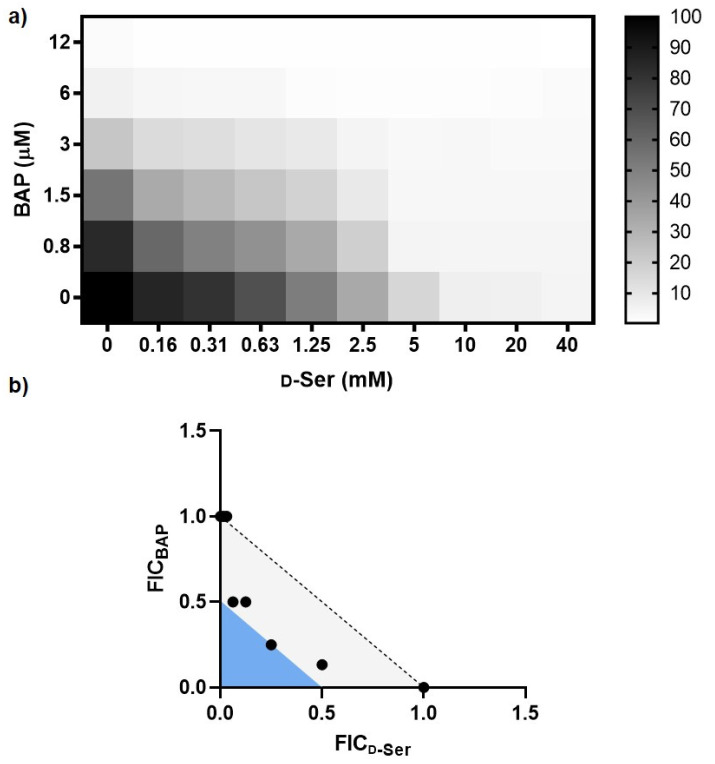
Combining BAP and d-Ser has an additive-to-synergistic effect against UPEC grown in MOPS-glycerol. (**a**) Representative checkerboard analysis with heat plot showing fractional growth at various combinations of BAP and d-Ser; (**b**) Isobologram analysis with points depicting FIC at fractional growth <10%; light gray region indicates additivity (FIC_I_ > 0.5 and <1.0), blue region indicates synergy (FIC_I_ ≤ 0.5). FIC_I_ were observed in the range 0.38–1.06 across experiments for all concentrations.

We hypothesized that DXPS activity should be essential for UPEC adaptation to d-Ser in the urinary tract since detoxification of d-Ser is mediated by the PLP-dependent deaminase DsdA, and PLP is biosynthesized from DXP. DXPS inhibition by BAP should limit the DsdA-dependent conversion of d-Ser, leading to its accumulation and bacteriostatic effects through inhibition of l-serine and pantothenate biosynthesis (Figure 1). Importantly, metabolic processes required for pathogen adaptation in vivo, such as UPEC adaptation to d-Ser, may not be evident under standard nutrient-rich culture conditions. Thus, we investigated this particular adaptation under nutrient-limitation conditions that are more relevant to infection in vivo [6,8,9,41,42,43]. A checkerboard analysis was conducted to evaluate the relationship between BAP and d-Ser, with the expectation that DXPS inhibition by BAP should enhance susceptibility of UPEC to d-Ser. The CFT073-WAM4505 (ATCC: BAA-2503) UPEC strain grown in MOPS minimal media containing 0.4% glycerol (MOPS-gly) was treated with a range of BAP and d-Ser concentrations in combination (Figure 2, Figure S1 from supplementary). Fractional inhibitory concentration index (FIC_I_) was determined for each combination. The results demonstrate a clear shift in potency of d-Ser in the presence of BAP, and an additive-to-synergistic relationship between BAP and d-Ser with FIC_I_ in the range of 0.375–1.03 across experiments. In contrast, the antimicrobial effect of d-Ser is not significantly enhanced by antibiotics with distinct mechanisms of action unrelated to d-Ser adaptation, including ampicillin and ciprofloxacin, which target cell wall biosynthesis and DNA replication, respectively (Figure S2 from supplementary). BAP-dependent sensitization of UPEC to d-Ser is consistent with a role of DXPS in this particular adaptation. 

### 2.2. β-Alanine Suppresses BAP Activity in the Presence of d-Ser

Additional evidence for a role of DXPS in adaptation to d-Ser was sought using metabolite suppression analysis, an approach that can reveal specific metabolic pathways targeted by antibiotics under nutrient-limitation [42]. Early studies to determine how d-Ser exerts its antimicrobial effects demonstrated suppression of d-Ser activity in the presence of β-alanine or pantothenate [32,33], indicating d-Ser inhibits pantothenate biosynthesis. Cosley and McFall showed that supplementation with l-serine suppresses d-Ser activity against a DsdA deletion mutant *E. coli* strain, suggesting d-Ser could also inhibit l-serine biosynthesis [33]. Specifically, d-Ser is thought to exert weak inhibitory activity against 3-phosphoglycerate dehydrogenase (SerA) which converts 3-phosphoglycerate (3-PGA) into 3-phosphohydroxypyruvate (3-PHP) in l-serine biosynthesis (Figure 1) [44], and aspartate 1-decarboxylase (PanD) which converts aspartate (Asp) to β-alanine (β-Ala), a precursor to pantothenate in the biosynthesis of CoA [45]. 

**Figure 3:**
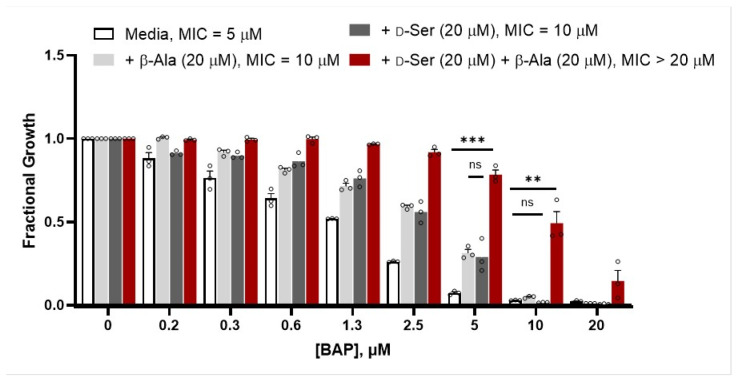
β-Alanine partially suppresses the activity of BAP against CFT073 in the presence of d-Ser; *p* > 0.05 (ns); *p* ≤ 0.01 (**); *p* ≤ 0.001 (***); n = 3, error bars represent standard error, data for all three replicates shown ().

We reasoned that if BAP-dependent inhibition of DXPS hinders d-Ser catabolism which, in turn, leads to inhibition of PanD in CFT073_wt_, then supplementation with β-Ala should partially suppress BAP-mediated growth inhibition in the presence of d-Ser. Indeed, at concentrations of BAP that suppress UPEC growth in the presence of d-Ser, addition of β-Ala partially rescues bacterial growth (Figure 3). This is consistent with a role of DXPS in d-ser catabolism. Partial rescue of UPEC growth by β-Ala in the presence of d-Ser is expected, since inhibiting DXP formation impacts multiple pathways, others of which cannot be rescued by the addition of β-Ala. Interestingly, at [BAP] < 10 μM, the fractional growth is higher for cultures supplemented with either β-Ala or d-Ser compared to unsupplemented cultures. The effects are subtle, increasing the MIC of BAP by only two-fold. Notably, this is only observed in the presence of BAP; supplementation of UPEC with either β-Ala or d-Ser does not confer an overall growth advantage in the absence of BAP. Analysis of the shifts in metabolic and/or regulatory networks upon treatment with BAP is required to fully understand these effects. Perhaps at lower [BAP], PLP is only weakly restricted and d-Ser is still catabolized to some extent, potentially boosting pyruvate concentrations to levels that could compete with BAP. On the other hand, weak restriction of PLP at low [BAP] could potentially hinder the rate-limiting step in pantothenate synthesis catalyzed by PanD, as PLP is required for conversion of OAA to the PanD substrate, Asp (Figure 1). Supplementation with β-Ala could relieve the stress of PLP restriction that limits Asp production. In d-Ser-treated cells exposed to higher [BAP], PLP restriction is expected to be more pronounced, causing d-Ser levels to increase and leading to a combined effect of PanD inhibition and reduced efficiency of Asp synthesis from OAA. In this condition, β-Ala supplementation is expected to have a more marked effect to rescue cell growth, as observed. 

### 2.3. DXPS Inhibition Leads to Increased DsdA Production in the Presence of d-Ser

**Figure 4:**
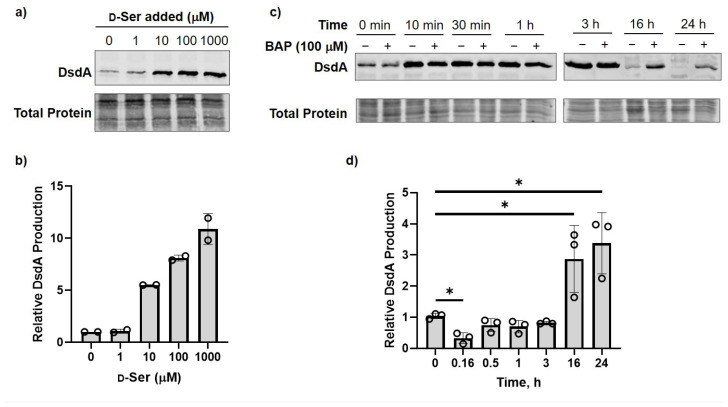
DsdA production in response to d-Ser, or BAP exposure in the presence of d-Ser. (**a**) Representative Western blot analysis showing that DsdA production increases in the presence of added d-Ser in MOPS-glycerol; (**b**) Quantified DsdA levels relative to 0 µM d-Ser added (*n* = 2, error bars represent standard deviation); (**c**) Representative Western blot analysis showing that enhanced, sustained DsdA production is observed in BAP-treated CFT073 over time; (**d**) Quantified DsdA levels in the presence of d-Ser under DXPS inhibition by BAP relative to the (-) BAP control at respective timepoints; *n* = 3, error bars represent standard deviation, data for all three replicates shown (). *p* > 0.05 (ns); *p* ≤ 0.05 (*).

Transcription of *dsdA* is under the control of the transcriptional regulator DsdC, which induces *dsdA* transcription in the presence of d-Ser, leading to increased DsdA activity in cell lysates [34,37]. Here, we confirmed DsdA protein production increases in the presence of d-Ser, with higher DsdA levels associated with increasing [d-Ser] in MOPS-glycerol, and an increase in DsdA levels observed in UPEC grown in urine compared to MOPS-glycerol lacking d-Ser (Figure 4a,b; Figure S3 from supplementary). 

Given that d-Ser induces production of DsdA in a concentration-dependent manner, we reasoned that increasing cellular d-Ser levels by inhibiting DXPS-dependent PLP synthesis should lead to higher levels of DsdA production. DsdA production levels in CFT073 treated with d-Ser were monitored over time in the presence or absence of BAP (Figure 4c,d). Upon addition of d-Ser, CFT073 respond rapidly (within 10 min) to increase DsdA production. DsdA levels are maintained presumably until d-Ser concentration drops to a non-toxic level (by 16 h). In the presence of BAP, DsdA levels are comparable to the (-) BAP control by 30 min (Figure 4d). BAP-dependent reduction in vitamin synthesis is expected to impair overall metabolism, including processes required for protein synthesis, which could explain the subtly slower increase in DsdA in BAP-treated cells relative to the (-) BAP control (Figure 4d). Higher DsdA levels are observed in the presence of BAP at longer timepoints (16 and 24 h), relative to the (-) BAP control (Figure 4c,d). This is consistent with our hypothesis that PLP synthesis is hindered as a result of DXPS inhibition, resulting in slower degradation of d-Ser. Prolonged d-Ser accumulation, in turn, induces DsdA production resulting in sustained higher levels of DsdA over a longer period of time. 

### 2.4. CFT073_ΔDsdA_ Is Hypersensitized to d-Serine under DXPS Inhibition

**Figure 5:**
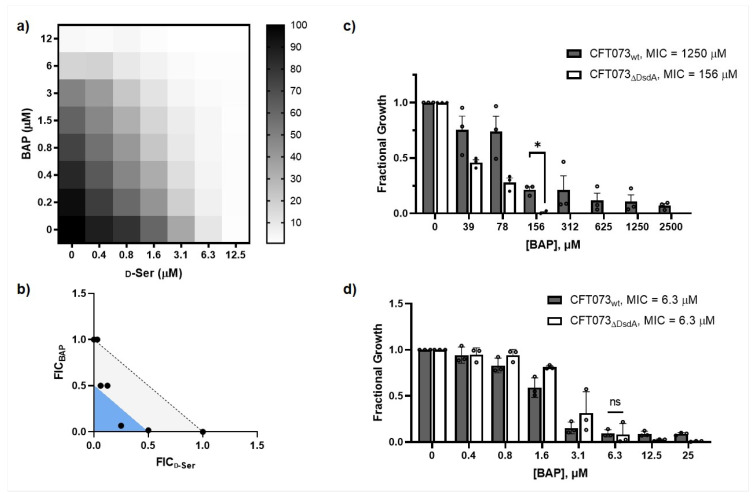
Combining BAP and d-Ser against CFT073_Δ DsdA_ grown in MOPS-glycerol shows an additive-to-synergistic relationship, and an increase in sensitivity to d-Ser relative to CFT073_wt_. (**a**) Representative checkerboard analysis showing a heat plot with fractional growth at various combinations of BAP and d-Ser; (**b**) Isobologram analysis with points depicting FIC at fractional growth <10%; light gray region indicates additivity (FIC_I_ > 0.5 and < 1.0), blue region indicates synergy (FIC_I_ ≤ 0.5), white region indicates no relationship. FIC_I_ were observed in the range 0.32–1.03 across experiments for all concentrations; (**c**) CFT073_ΔDsdA_ sensitization to BAP in pooled human urine (MIC = 156 μM) compared to CFT073_wt_ (MIC = 1250 μM), *n* = 3, error bars represent standard error; (**d**) CFT073_ΔDsdA_ is not sensitized to BAP in MOPS-gly compared to CFT073_wt_ (MIC = 6.3 μM), *n* = 3; error bars represent standard error; data for all three replicates shown (); *p* > 0.05 (ns); *p* ≤ 0.05 (*).

The relationship between BAP and d-Ser in CFT073 (Figure 2) not only supports a role of DXPS in adaptation to d-Ser, but also suggests that inhibiting DXPS creates a metabolic vulnerability that sensitizes UPEC to inhibition of l-Ser and/or CoA biosynthesis. The toxic effects of d-Ser can be leveraged to investigate this metabolic vulnerability. To this end, we assessed the relationship and potency of the BAP/d-Ser combination against a DsdA deletion mutant of CFT073 (CFT073_ΔDsdA_) which is unable to catabolize d-Ser [46]. An additive-to-synergistic effect of the BAP/d-Ser combination against CFT073_ΔDsdA_ and increased potency of this combination compared to CFT073_wt_ is observed in MOPS-gly (Figure 5a,b, Figure S4 from supplementary). CFT073_ΔDsdA_ is also sensitized to DXPS inhibition by BAP in d-Ser-containing pooled human urine compared to CFT073_wt_ (eight-fold shift in MIC), but not in MOPS-glycerol (Figure 5c,d). These results suggest a metabolic vulnerability caused by DXPS inhibition specifically in the context of UTI which could be exploited in the design of prevention or treatment strategies for UTI.

### 2.5. BAP-Treated CFT073 Grown in Urine Are Sensitized to Inhibition of CoA Synthesis

As noted, CFT073_ΔDsdA_ cells are incapable of catabolizing d-Ser, resulting in unrestricted d-Ser-mediated inhibition of l-serine and CoA synthesis. The positive relationship between BAP and d-Ser (Figure 5a,b) and the sensitization of CFT073_ΔDsdA_ to BAP when grown in urine (Figure 5c) suggest strategies for combination therapy that UPEC could be especially sensitive to within the urinary tract. For example, we reasoned that d-Ser-mediated inhibition of CoA biosynthesis should be particularly problematic for UPEC under conditions of DXPS inhibition during UTI. UPEC rely heavily on a functioning TCA cycle and gluconeogenesis for survival in the urinary tract [25]; introducing multiple bottlenecks to limit the ThDP-dependent conversion of coenzyme A (CoA) to acetyl-CoA, as well as the PLP-dependent production of pyruvate (Pyr) and oxaloacetate (OAA), should critically impair the ability of UPEC to replenish the TCA cycle and provide essential precursors for gluconeogenesis. Thus, combining inhibitors of DXPS and CoA synthesis could be potent in the context of UTI. 

**Figure 6:**
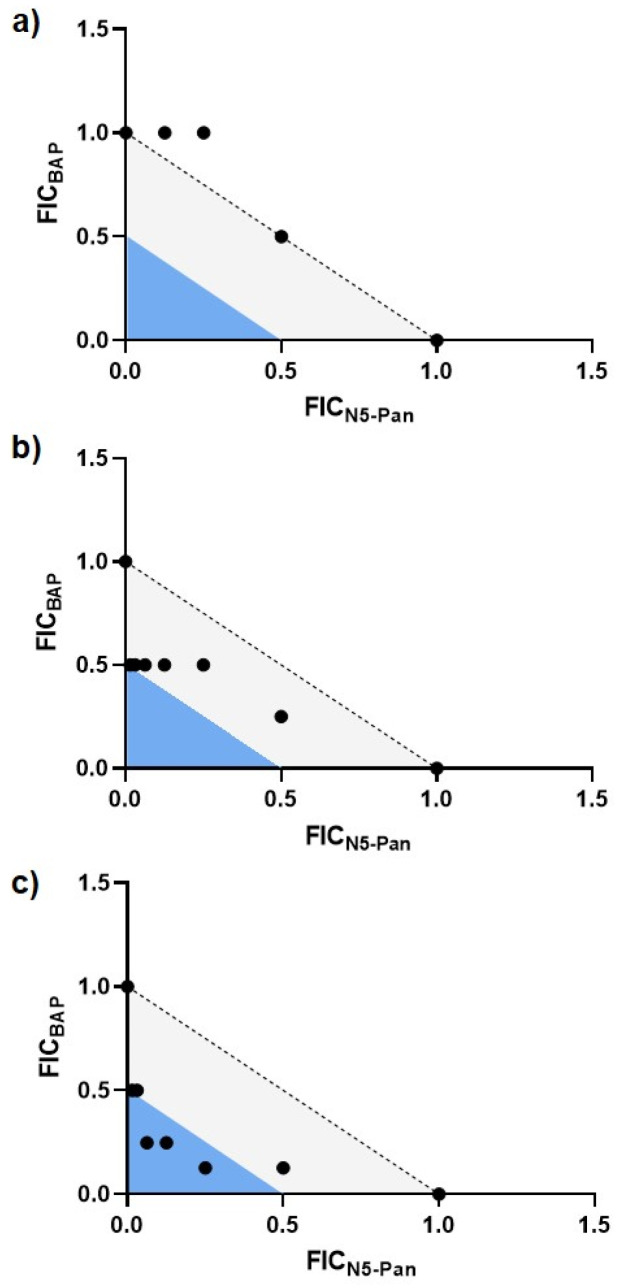
Growth medium effects on the BAP/N5-Pan relationship. Additive or additive-to-synergistic effects are observed in growth conditions where UPEC possess an increased reliance on TCA cycle gluconeogenesis from amino acids. Representative isobologram analyses are shown with points depicting FIC at fractional growth <10% growth; white region indicates indifference (FIC_I_ > 1.0), light gray region indicates additivity (FIC_I_ > 0.5 and <1.0), blue region indicates synergy (FIC_I_ ≤ 0.5). (**a**) Isobologram analysis showing additive-to-indifferent relationship in MOPS-glycerol with FIC_I_ observed in the range 0.75–2.00 across experiments for all concentrations; (**b**) Isobologram analysis showing primarily an additive relationship in M9-casamino acids with FIC_I_ observed in the range 0.5–1.03 across experiments for all concentrations; (**c**) Isobologram analysis showing additive-to-synergistic relationship in urine with FIC_I_ observed in the range 0.25–1.01 across experiments for all concentrations.

CoA is an essential cofactor, playing a vital role in the TCA cycle as well as other essential metabolic processes in bacteria. Impairing CoA biosynthesis as an antibacterial strategy has been explored [45,47,48,49,50,51], and the antimetabolite N5-Pan (*N*-pentyl pantothenamide) is known to inhibit *E. coli* growth by reducing cellular CoA. N5-Pan is thought to exert its potent effects against *E. coli* through inhibition of the rate-limiting phosphorylation of pantothenate (CoaA), and conversion to a CoA analog, EtdtCoA, which in turn acts to downregulate pantothenate synthesis mediated by the PanDZ complex [47,48,49,50,51,52]. We hypothesized that combining N5-Pan and BAP may more potently impair UPEC growth in M9-casamino acids (M9-CasAA) or urine where UPEC rely heavily on PLP- and ThDP-dependent processes for the TCA cycle and gluconeogenesis from amino acids, and where d-Ser could accumulate to toxic levels under DXPS inhibition (urine specifically). Checkerboard analysis of the N5-Pan/BAP combination against CFT073 grown in MOPS-glycerol shows an additive-to-indifferent relationship (Figure 6a, Figure S5 from supplementary). However, combining BAP and N5-Pan against CFT073 shows an additive relationship in M9-CasAA (Figure 6b, Figure S6 from supplementary), and an additive-to-synergistic relationship in urine (Figure 6c, Figure S7 from supplementary). Thus, BAP-treated UPEC appear to be more vulnerable to inhibition of CoA synthesis when catabolism of amino acids is required. 

## 3. Discussion

Pathogenic bacteria are exposed to fluctuating host environments during infection, which impose unique metabolic requirements to which the pathogen must adapt. Uropathogens originating from the gut experience a dramatic change in nutrient availability as they transition to the urinary tract. Urine flow coupled with the host immune response inflict further obstacles that UPEC must successfully overcome. UPEC are highly responsive to changes in their environment; thus, despite the nutrient-limiting conditions and harsh environment of the urinary tract, UPEC efficiently adapt by remodeling metabolism, colonizing the urinary tract and causing infection. Developing agents that impair these adaptations and sensitize uropathogens to the stressful environment of the urinary tract represent a promising strategy to prevent or treat UTI.

This study investigated a branchpoint enzyme in bacterial central metabolism, DXPS, as an intriguing target to investigate the effects of metabolic impairment on uropathogen adaptation. UPEC adapt to utilize peptides and amino acids as the main carbon source in urine. The specific adaptation to d-Ser studied here involves upregulation of the PLP-dependent enzyme DsdA to both detoxify d-Ser and utilize it as a carbon source. By investigating the toxic effects of d-Ser as it accumulates in the presence of a DXPS inhibitor, we have demonstrated a role of DXPS in this PLP-dependent adaptation to d-Ser. Inhibition of DXPS sensitizes UPEC to d-Ser, with BAP and d-Ser exhibiting a synergistic-to-additive relationship. This BAP-mediated toxicity to UPEC in the presence of d-Ser is partially suppressed when CFT073_wt_ are supplied with β-Ala, the product of the d-Ser-targeted enzyme PanD. Further, consistent with a downstream effect of DXPS inhibition to impede d-Ser catabolism, DsdA levels are sustained at higher levels for a longer period of time in d-Ser-treated cells in response to BAP exposure.

There is more pronounced synergy in the synergistic-to-additive effect of combining BAP and d-Ser against CFT073_ΔDsdA_, a UPEC strain incapable of catabolizing d-Ser. CFT073_ΔDsdA_ is also hypersensitized to BAP in urine, but not in MOPS-glycerol. These results point to a metabolic vulnerability caused by inhibiting this branch point that can potentially be exploited for development of UTI-specific treatment or prevention strategies. Specifically, we tested the idea that combining inhibitors of DXP and CoA production should potently inhibit uropathogen growth in urine, given that UPEC heavily rely upon PLP-dependent catabolic processes and a functional TCA cycle for gluconeogenesis in this environment. Indeed, BAP and N5-Pan, an inhibitor of CoA biosynthesis, display a synergistic-to-additive relationship against CFT073 grown in urine and primarily additivity in M9-casamino acids. In contrast, the BAP/N5-Pan combination displays an additive-to-indifferent effect when CFT073 are grown in MOPS-glycerol. Thus, the increased reliance on acetyl-CoA production and the TCA cycle for gluconeogenesis from amino acids appears to render BAP-treated UPEC more vulnerable to inhibition of CoA synthesis. Inhibition of early and late-stage CoA biosynthesis (PanD & CoaA), as well as weak inhibition of l-serine biosynthesis, by the combined action of N5-Pan and accumulated d-Ser, which is present in urine but not in casamino acids, could contribute to the observed synergy in urine. It is also plausible that restricted access to some amino acids in urine could further enhance sensitivity to inhibition of DXPS and CoA synthesis in this condition. Thus, conditional susceptibility to inhibition of CoA biosynthesis under conditions of DXPS inhibition specifically in the urinary tract may provide a UPEC-specific antibacterial strategy. Given that BAP is a relatively weak antimicrobial agent on its own against UPEC grown in urine, these results also offer some promise for BAP as an antimicrobial agent when used in combination therapy.

DXPS-dependent adaptation to d-Ser is the first to be characterized, but almost certainly not the only adaptation of UPEC (or other pathogens) that relies upon DXPS activity. DXPS sits at a metabolic branchpoint; thus, we expect its inhibition could broadly impair the adaptation of uropathogens within the urinary tract. Although not studied here, impeding isoprenoid precursor production via DXPS inhibition may also critically impact the ability of UPEC to metabolically adapt during UTI. For example, limiting isoprenoid synthesis is expected to impair ATP generation by disrupting the electron transport chain. Given that ATP synthesis is required for UPEC fitness in urine [53,54], this could represent an additional metabolic vulnerability of UPEC in the face of DXPS inhibition. Further, bacterial metabolism and pathogenicity are known to be closely linked [1,55,56]; thus, interfering with metabolism through inhibition of DXPS might also have the potential to limit pathogen virulence.

Finally, this work underscores the importance of developing antibacterial strategies in the context of the particular pathogen and its relevant host environments. Growth environment is an especially important consideration for the study of central metabolic inhibitors such as BAP. Conventional nutrient-rich culture conditions have the potential to abrogate the essentiality of some otherwise in vivo-essential metabolic pathways. Infection sites in vivo limit nutrient access for pathogens forcing them to synthesize key metabolites from limited resources. Our studies demonstrate how urine as a growth condition can impact the antimicrobial potency of a DXPS inhibitor against UPEC, as well as DXPS inhibitor relationships in the design of UTI-specific combination therapies. Considering the many roles of the microbiome in human health, it is becoming increasingly important to invent pathogen-specific treatments that avoid toxicity to healthy microbiota. Inhibition of DXPS-dependent pathogen-specific adaptations may be a potential avenue to narrow-spectrum strategies to combat antibacterial resistance.

## 4. Methods

### 4.1. General Methods

Unless otherwise noted, reagents were obtained from commercial sources. BAP and N5-Pan were synthesized according to previously published methods [49,52,57,58,59,60], with modifications to N5-Pan synthesis detailed in Supporting Information. Pooled human urine was obtained from Innovative Research, Inc. (Novi, MI, USA) or Lee BioSolutions, Inc. (Maryland Heights, MO, USA) and filter-sterilized prior to use. Solid MOPS-glycerol agar plates were prepared by supplementing cooled 1.5% (*w*/*v*) agar with 10 × MOPS Buffer (Teknova, Half Moon Bay, CA, USA) to a final 1 × concentration (pH 7.4), 1.32 mM K_2_HPO_4_ and 0.4% (*v*/*v*) glycerol. Strains were streaked from glycerol stocks onto agar plates and incubated at 37 °C, 48 h. Liquid MOPS-glycerol was prepared as above, without added agar, by adding 10 × MOPS Buffer (Teknova) to autoclaved water to a final 1 × concentration, 1.32 mM K_2_HPO_4_ and 0.4% (*v*/*v*) glycerol. Solid urine agar plates were prepared by supplementing cooled 1.5% (*w*/*v*) agar with filter sterilized urine in a 4:1 ratio. OD_600_ and Bradford analyses were performed on Beckman DU800 UV/Visible spectrophotometer (Brea, CA, USA). Antimicrobial data were collected on a Biotek Epoch 2 microplate reader, a Biotek Synergy Neo 2 microplate reader, or a Tecan Infinite M200 Nanoquant plate reader. Uropathogenic *E. coli* strains CFT073 (ATCC: BAA-2503) and CFT073_ΔDsdA_ were provided by Kevin Schwartz from the lab of Rodney Welch. All microbial manipulation of pathogenic bacteria was conducted in a certified biosafety level 2 laboratory while following all associated safety protocols.

### 4.2. Antimicrobial Susceptibility Studies

Using aseptic technique, CFT073 from frozen glycerol stocks were streaked onto MOPS-glycerol agar plates and allowed to grow for 48 h at 37 °C. Three to five isolated colonies were inoculated into liquid MOPS-glycerol (3 mL) supplemented with 20 µM FeSO_4_, and the resulting culture was grown overnight with shaking at 37 °C. The overnight cultures were then subcultured (1:50) and grown to exponential phase (OD_600_ = 0.4). Cultures at exponential phase were diluted 1:1000 into fresh MOPS-glycerol medium to yield the experimental inoculum which was mixed 1:1 with MOPS-glycerol medium containing BAP, d-Ser or N5-Pan at 2× the desired final concentration. The final concentration of bacteria in each well was approximately 10^5^ CFU/mL in a final volume of 200 µL. Colony counts of the experimental inoculum were independently verified by dilution and enumeration on CAMHB agar for 16 h at 37 °C to confirm consistency between experiments. The 96-well plates were incubated at 37 °C for 24 h with intermittent shaking. Fractional growth of drug-treated cells was determined at 24 h relative to the no drug control. Experiments were performed in biological triplicate.

For antimicrobial susceptibility studies conducted in pooled urine, a similar approach was taken. CFT073 from frozen glycerol stocks were streaked onto urine agar plates and allowed to grow for 48 h at 37 °C. Three to five isolated colonies were inoculated into filter-sterilized urine supplemented with 20 µM FeSO_4_ and grown to saturation overnight with shaking at 37 °C. The saturated cultures were then subcultured (1:50) and grown to exponential phase (OD_600_ = 0.25). Cultures at exponential phase were diluted 1:1000 into fresh medium to yield the experimental inoculum which was mixed 1:1 with urine containing the antimicrobial agent at 2× the desired final concentration. The final concentration of bacteria in each well was approximately 10^5^ CFU/mL in a final volume of 200 µL. Colony counts of the experimental inoculum were independently verified by dilution and enumeration on CAMHB agar for 16 h at 37 °C to confirm consistency between experiments. The 96-well plates were incubated at 37 °C for 16 h with intermittent shaking. Fractional growth of drug-treated cells was determined at 8 h relative to the no drug control. Experiments were performed in biological triplicate.

### 4.3. Checkerboard Analysis

An initial inoculum of 10^5^ CFU/mL CFT073 was prepared as described above, in MOPS-glycerol, M9 with 0.4% casamino acids or pooled human urine. Cells were combined at varied concentrations of BAP and d-Ser in combination (0–12 µM BAP, 0–40 mM d-Ser in MOPS-glycerol), or BAP and N5-Pan in combination (MOPS-glycerol: 0–12 µM BAP, 0–312 µM N5-Pan; casamino acids: 0–312.5 µM BAP, 0–312.5 µM N5-Pan; urine: 0–1250 µM BAP, 0–312 µM N5-Pan) in a 96-well plate, as previously described [17,61] (and incubated at 37 °C for 24 h (MOPS-glycerol) or 16 h (urine). Cell growth was measured by absorbance, and fractional growth was calculated relative to the no drug control at 24 h (MOPS-glycerol and casamino acids), or 8 h (urine). Experiments were performed in biological and technical triplicate.

Fractional inhibitory concentration indices (*FIC_I_*) were determined as previously described [62,63]). Briefly, *FIC_I_* were calculated using Equation (1).

(1) $$FIC\;index=FIC_{A}+FIC_{B}=\left(\frac{\left[A\right]}{\begin{array}{c} MIC_{A} \end{array}}\right)+\left(\frac{\left[B\right]}{\begin{array}{c} MIC_{B} \end{array}}\right)$$

*MIC_A_* and *MIC_B_* represent the lowest concentrations of *A* or *B*, respectively, required to inhibit bacterial growth by 90%. *FIC_A_* was calculated as [*A*] in the presence of *B* for a well showing <10% growth, divided by *MIC_A_*. *FIC_B_* was calculated as [*B*] in the presence of *A* for a well showing <10% growth, divided by *MIC_B_*. The *FIC* index (*FIC_I_* is the sum of individual *FICs* for *A* and *B*. *FIC_I_* were used to indicate synergy (≤0.5), additivity (≥0.5 and <1) or indifference (>1 and <2). Isobolograms were constructed from the checkerboard analysis by plotting *FIC* values determined for each compound (*FIC_A_* and *FIC_B_*) over the range of Drug A concentrations tested in combination with Drug B (see Figure S8 from supplementary). Points falling within the light gray region of the isobologram indicate additivity. Points falling within the blue region or at the interface of blue and gray regions indicate synergy. Points falling above the line of additivity indicate indifference.

### 4.4. Metabolite Suppression Analysis

Experiments were performed according to the procedure described above for antimicrobial susceptibility with the following modification: cultures at exponential phase were diluted 1:1000 into fresh medium to yield the experimental inoculum which was mixed 1:1 with MOPS-glycerol medium containing the antimicrobial agent at 2× the desired final concentration, plus β-alanine in the presence or absence of d-Ser to give β-alanine and d-Ser at final concentrations of 20 µM, respectively, in each well.

### 4.5. DsdA Expression Analysis

Using aseptic technique, three to five isolated colonies were picked from a MOPS-glycerol or urine agar plate and inoculated into liquid MOPS-glycerol or urine. FeSO_4_ (20 µM) was added immediately prior to inoculation. Cultures were grown to saturation overnight with shaking at 37 °C. The saturated cultures were subcultured (1:50) into 80 mL of fresh medium and grown to exponential phase as measured by absorbance (O.D. ~ 0.4 for cultures in MOPS-glycerol; O.D. ~ 0.25 for cultures in urine). Cells were then treated with d-Ser or d-Ser + BAP at a final concentration of 100 µM of each. Aliquots of cultures were removed at indicated timepoints, centrifuged 4000× *g* for 20 min, then resuspended in lysis buffer (50 mM TBS + 1 × PIC + 1 mM PMSF, pH 7.2). Resuspended samples were then sonicated to lyse cells (Branson Sonifier SFX250; Danbury, CT, USA). Crude cell lysates were centrifuged at 20,000× *g* for 10 min, and the supernatant was collected and quantified for total protein content by Bradford reagent. A volume of lysate for each sample (5–15 μL) containing 15 µg of protein was mixed with 5× SDS-loading dye and analyzed by SDS-PAGE (10% SDS-PAGE), followed by semi-dry transfer (Pierce Power System, Waltham, MA, USA) to a nitrocellulose membrane. Total protein transferred was quantified with Revert 700 Total Protein Stain (Li-Cor; Lincoln, NE, USA). The blot was developed using protein-specific polyclonal anti-DsdA (LSBio; Seattle, WA, USA) as primary antibody and IRDye 800 CW goat anti-rabbit IgG as secondary antibody (Li-Cor; Lincoln, NE, USA). Total protein levels, as determined by Revert 700 staining, were quantified with Image Studio (Li-Cor; Lincoln, NE, USA). DsdA level was normalized based on the total protein levels after transfer in each lane and is reported relative to the untreated control at each time point. Experiments were performed in biological and technical triplicate.

### 4.6. Statistical Analyses

Statistical analyses were performed using Graph Pad Prism. Statistical difference of measured FIC_I_ to the additive model (FIC_I_ = 1) was assessed using Wilcoxon signed rank tests (Figure S9 from supplementary).

## Data Availability

All data are contained within this article.

## Supplementary Materials

The following supporting information can be downloaded at: https://www.mdpi.com/article/10.3390/antibiotics12040692/s1, Figure S1: Replicate checkerboards and isobologram analyses for the BAP/d-Ser combination in MOPS-glycerol. Figure S2: Checkerboards for d-Ser/Cipro and d-Ser/Amp. Figure S3: DsdA production analysis. Figure S4: Replicate checkerboards and isobologram analyses for the BAP/d-Ser combination CFT073_ΔDsdA_. Figure S5: Replicate checkerboards and isobologram analyses for the BAP/N5-Pan combination in MOPS-glycerol. Figure S6: Replicate checkerboards and isobologram analyses for the BAP/N5-Pan combination in M9-CasAA. Figure S7: Replicate checkerboards and isobologram analyses for the BAP/N5-Pan combination in pooled human urine. Figure S8: Method for assembling isobolograms. Figure S9: Isobolograms, averaged FIC values.
