# Supplementary material for: DXP Synthase Function in a Bacterial Metabolic Adaptation and Implications for Antibacterial Strategies
Source: Antibiotics (Basel). 2023 Apr 1;12(4):692. doi: 10.3390/antibiotics12040692 (PMC10135061; doi:10.3390/antibiotics12040692)
Supplement: Supplementary file 1 [file antibiotics-12-00692-s001.zip › antibiotics-2284947-supplementary.pdf]

DXP Synthase Function in a Bacterial Metabolic Adaptation and Implications for Antibacterial Strategies

Eric C. Chen and Caren L. Freel Meyers *

Department of Pharmacology and Molecular Sciences, The Johns Hopkins School of Medicine, Baltimore, MD 21201, USA
* Correspondence: cmeyers@jhmi.edu.

Table of Contents

page

Synthesis of *N*-pentyl pantothenamide (N5-Pan)……………………………………………………2

Figure S1: Replicate checkerboards and isobologram analyses for the BAP/D-Ser combination in MOPS-glycerol……………………………………………………………………………………3

Figure S2: Additive-to-indifferent relationship of D-Ser/Cipro and D-Ser/Ampicillin……………….4

Figure S3: DsdA production analysis………………………………………………………………..5

Figure S4: Replicate checkerboards and isobologram analyses showing an additive-to-synergistic relationship of BAP/D-Ser……………………………………………………………6

Figure S5: Replicate checkerboard and isobologram analyses for BAP/N5-Pan combination studies in MOPS-glycerol………………………………………………………………………...7

Figure S6: Replicate checkerboard and isobologram analyses for BAP/N5-Pan combination studies in M9-CasAA……………………………………………………………………………….8

Figure S7: Replicate checkerboard and isobologram analyses for BAP/N5-Pan combination studies in pooled human urine………………………………………………………………………..9

Figure S8: Method for assembling isobolograms…..………………………………………………..10

Figure S9: Isobolograms, averaged FIC values ……………………………………………………11

Synthesis of *N*-pentyl pantothenamide (N5-Pan)

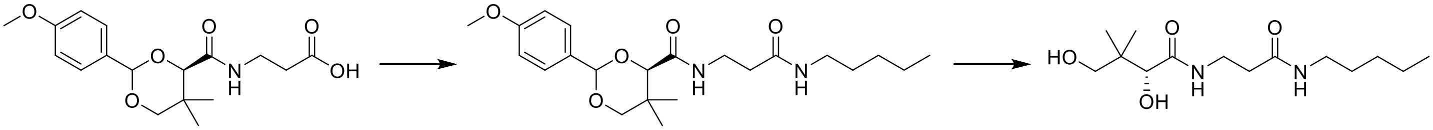

1 2 **N5-Pan**

3-((4R)-2-(4-methoxyphenyl)-5,5-dimethyl-1,3-dioxane-4-carboxamido)propanoic acid (1) was synthesized following a procedure described by Patrone, J.D., et al [1].

Synthesis of (4R)-2-(4-methoxyphenyl)-5,5-dimethyl-N-(3-oxo-3-(pentylamino)propyl)-1,3-dioxane-4-carboxamide (2): To a solution of **1** (1075 mg, 3.19 mmol, 1.0 equiv.) in anhydrous DMF (0.5 M, 6.4 ml), was added *N,N*-diisopropylethylamine (0.83 ml, 4.78 mmol, 1.5 equiv.) followed by HATU (1242 mg, 3.265 mmol, 1.025 equiv.) in one portion at 0°C. After stirring at 0°C for ca. 2-3 min, 1-aminopentane (0.74 ml, 6.37 mmol, 2.0 equiv.) was added in one portion, and the ice/water bath was removed. After stirring at rt for 18h, the reaction mixture was diluted with water, ethyl acetate and ca. 2 ml sat. aq. Na_2CO_3. The mixture was extracted with EtOAc (3 x 20 ml), and the organic layers were combined, washed with brine and dried over anhydrous $MgSO_4$. The volatiles were removed *in vacuo*, and the resulting residue was absorbed onto silica gel and purified via silica gel flash chromatography (gradient 0-100% hexanes/(79% ethyl acetate, 20% methanol, 1% Et_3N)) to provide 1.17 g of white solid (82% yield). Rf=0.7 (100% EtOAc). ^{1}H NMR (500 MHz, CHLOROFORM-d) δ 7.34 - 7.52 (m, 2H), 7.01 (t, *J* = 5.50 Hz, 1H), 6.84 - 6.96 (m, 2H), 5.78 (br. s., 1H), 5.46 (s, 1H), 4.08 (s, 1H), 3.83 (s, 3H), 3.62 - 3.76 (m, 2H), 3.42 - 3.62 (m, 2H), 3.14 - 3.30 (m, 2H), 2.41 (t, *J* = 6.21 Hz, 2H), 1.47 (quin, *J* = 7.31 Hz, 2H), 1.19 - 1.39 (m, 4H), 1.10 (d, *J* = 1.89 Hz, 6H), 0.89 (t, *J* = 7.07 Hz, 3H).

Synthesis of (R)-2,4-dihydroxy-3,3-dimethyl-N-(3-oxo-3-(pentylamino)propyl)butanamide (N-pentyl pantothenamide or N5-Pan) was synthesized following a procedure described by Awuah, E., et al. (Supporting Information. General procedure C: Deprotection of PMB-protected N-substituted pantothenamides)[2]. Compound **2** (1175 mg, 2.89 mmol) was dissolved in 10 ml of AcOH:H_2O (9:1). The reaction mixture was stirred at room temperature until the disappearance of the starting material was observed (ca. 16 h). Silica gel was added to the complete reaction, and the volatiles were removed *in vacuo*. The resulting residue was purified via silica gel flash chromatography (gradient 0-20% dichloromethane/MeOH) to provide a white solid which was further purified by recrystallization from boiling EtOAc, and stored at -20°C. The characterization data were consistent with that previously reported [3].

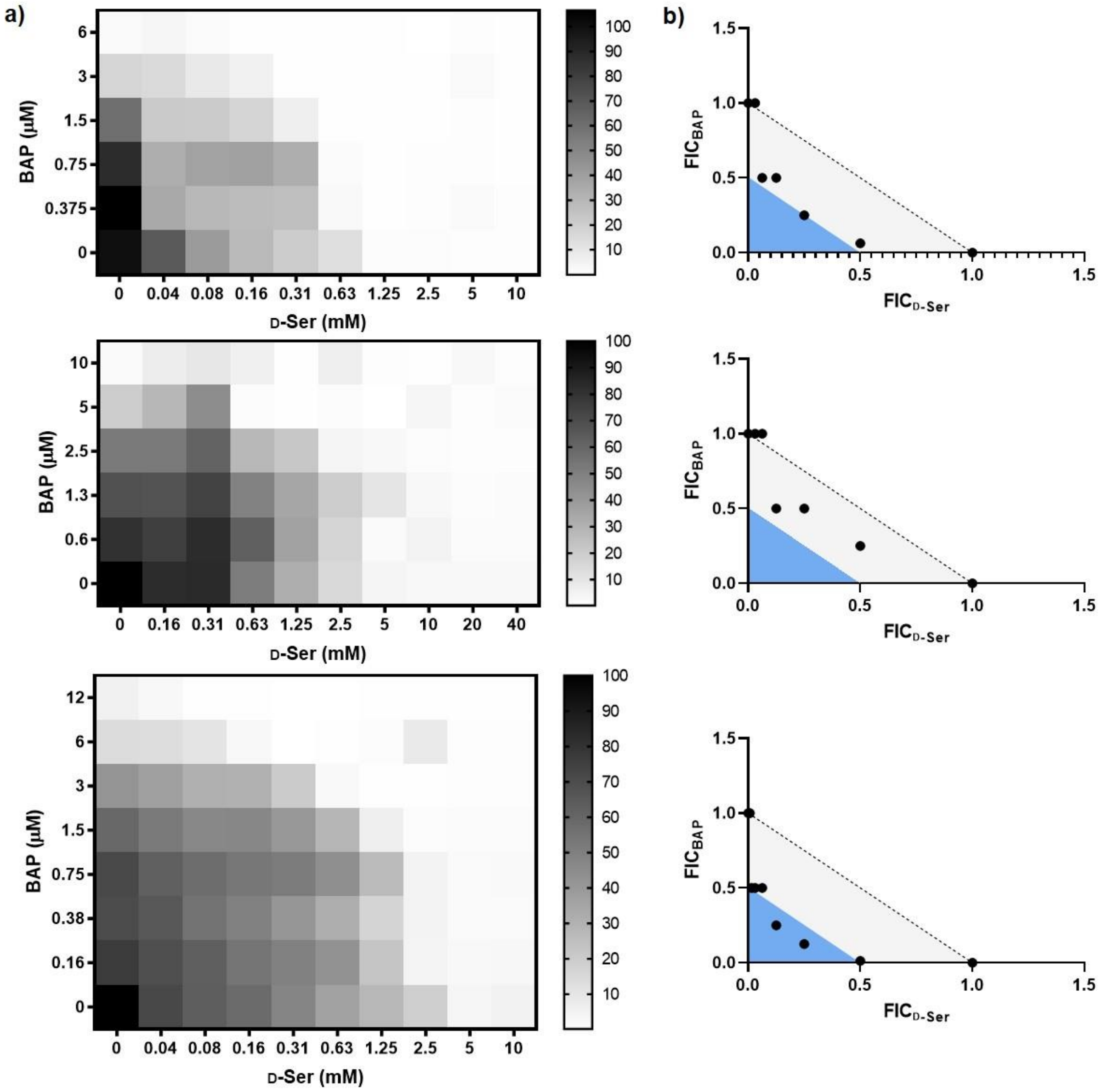

Figure S1. Replicate checkerboards and isobologram analyses for the BAP/D-Ser combination in MOPS-glycerol show an overall additive-to-synergistic effect. **a)** Replicate checkerboard analyses with heat plots showing fractional growth at various combinations of BAP and D-Ser; **b)** Corresponding replicate isobolograms (see Fig S8 for explanation); light gray region indicates additivity (FIC_I > 0.5 and < 1.0), blue region indicates synergy ($FIC_I \leq 0.5$). FIC_I were observed in the range 0.38 – 1.06 across experiments for all concentrations.

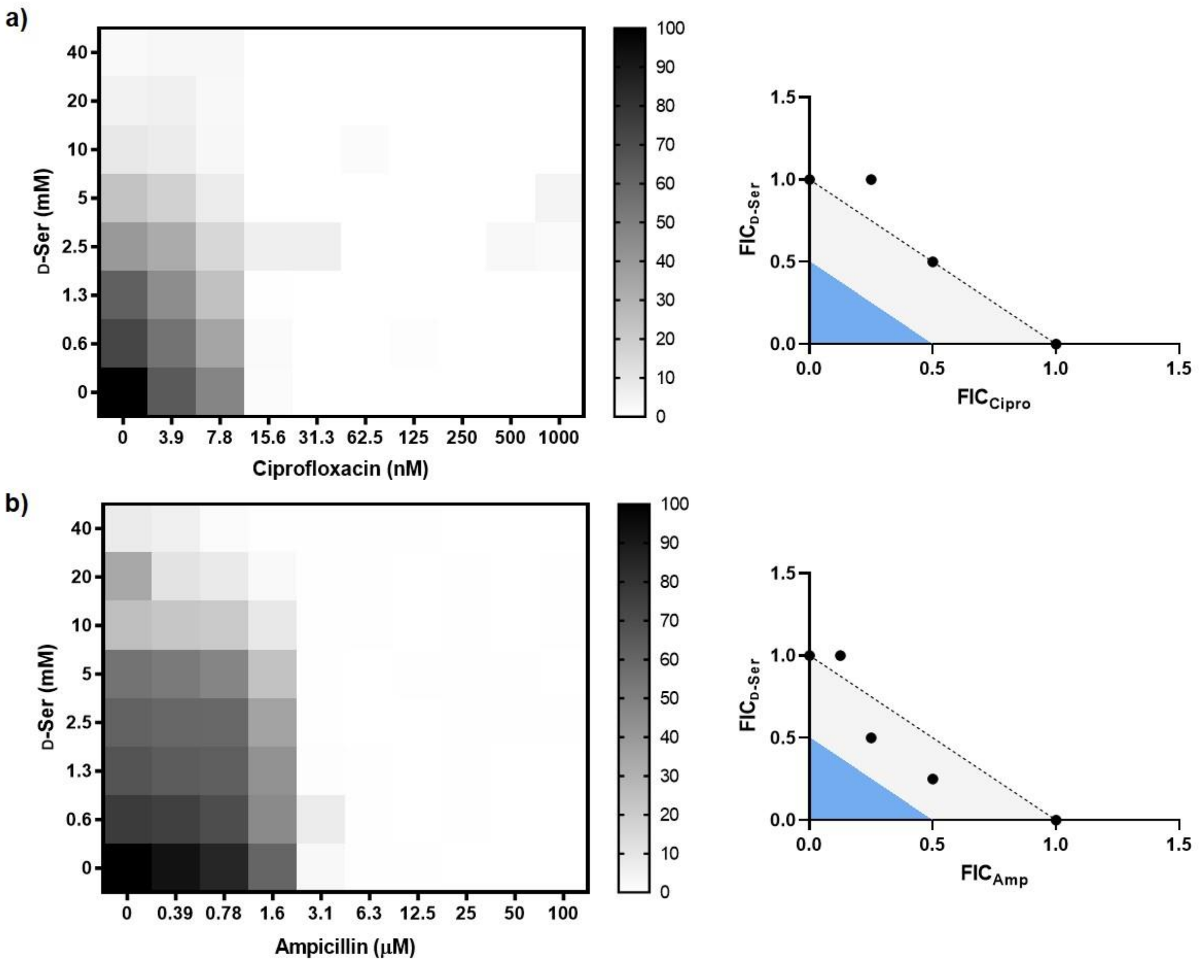

Figure S2. Combining D-Ser with other antibiotics has an additive-to-indifferent effect against UPEC grown in MOPS-glycerol. **a)** Checkerboard analyses with heat plots showing fractional growth at various combinations of D-Ser and ampicillin or ciprofloxacin **b)** Corresponding Isobologram analysis (see Fig S8 for explanation); light gray region indicates additivity (FIC_I > 0.5 and < 1.0), blue region indicates synergy ($FIC_I \leq 0.5$). FIC_I were observed in the range 1.0 – 1.25 for ciprofloxacin and 0.75 – 1.13 for ampicillin.

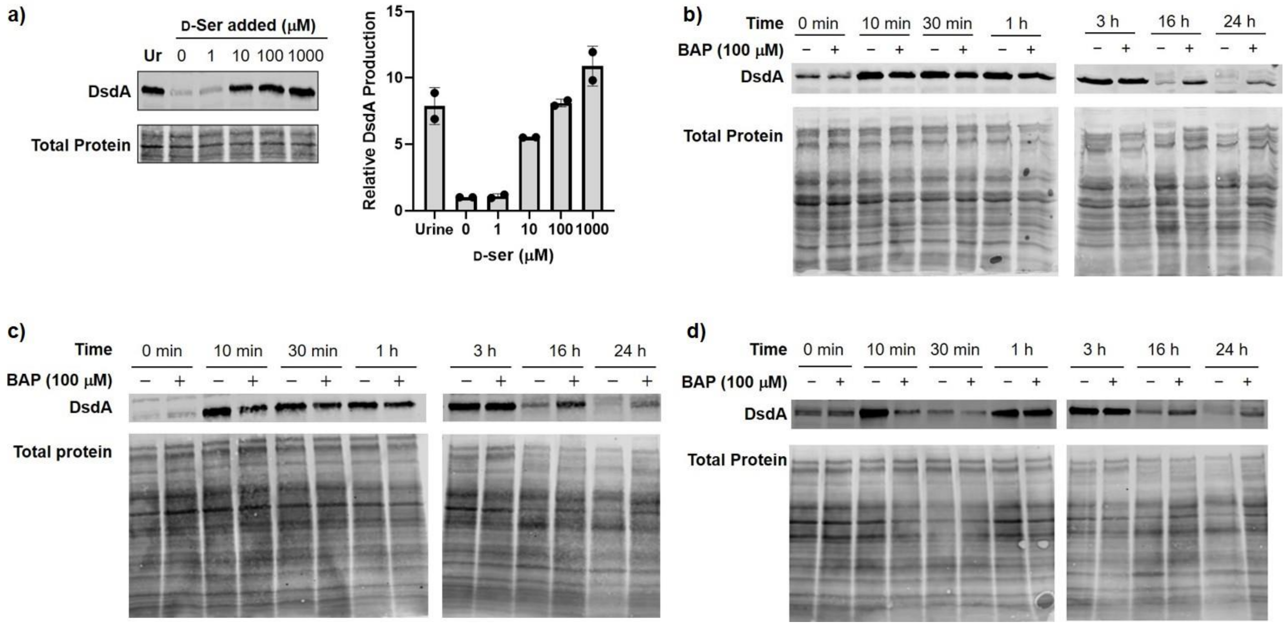

Figure S3. DsdA production analyses. **a)** DsdA production is elevated in urine (labelled "Ur") relative to MOPS-gly (n = 2, error bars represent standard deviation). DsdA production also increases in the presence of added D-Ser when CFT073 are grown in MOPS-gly (replicate of Fig 4a,b); **b)** Western blot analysis in Figure 4c with total protein gel shown; **c-d)** Replicate western blot analysis showing enhanced, sustained DsdA production in BAP-treated CFT073 over time, normalized to total protein.

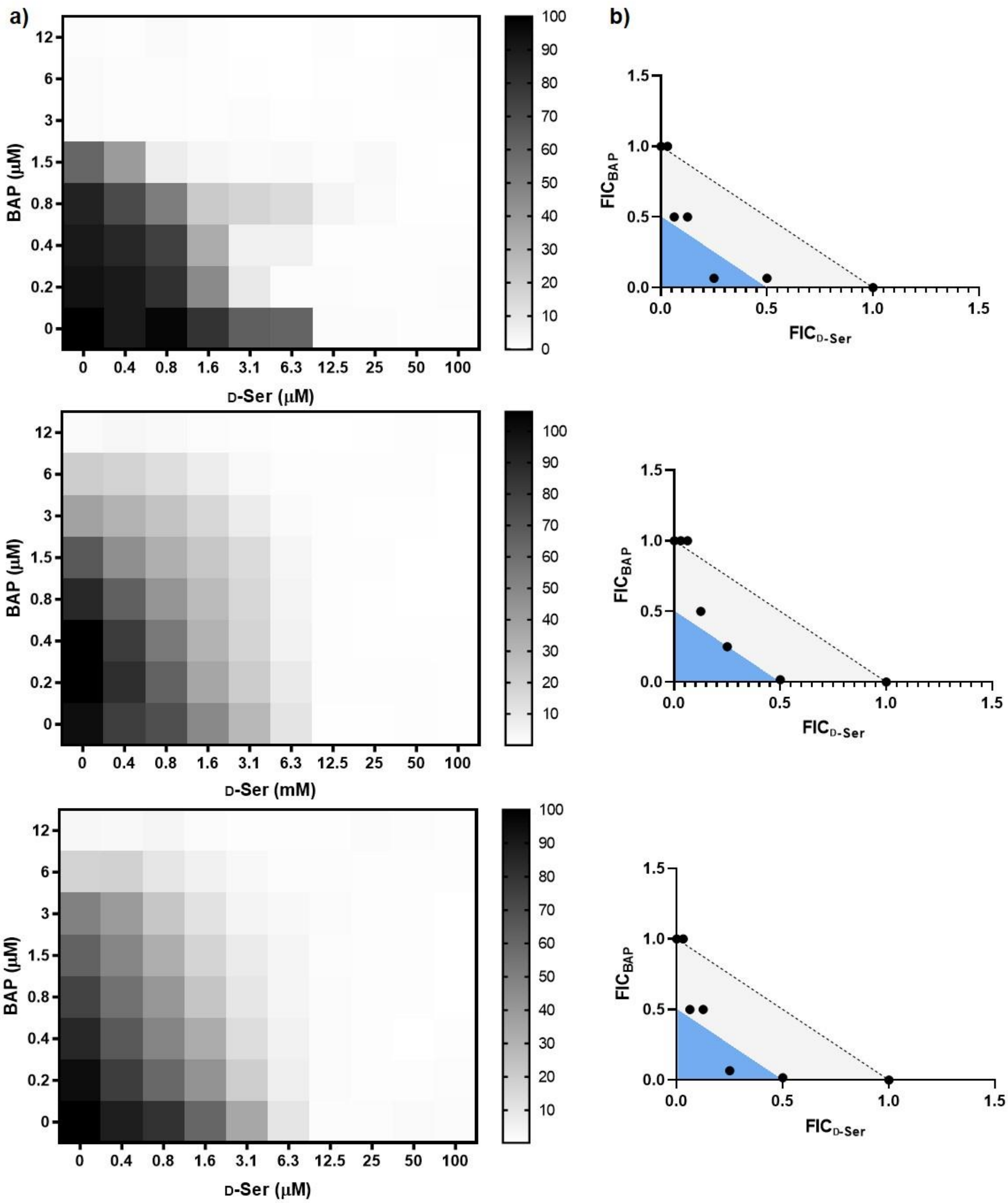

Figure S4. Replicate checkerboards and isobologram analyses of $CFT073_{\Delta DsdA}$ grown in MOPS-glycerol showing an additive-to-synergistic relationship of BAP/D-Ser. **a)** Replicate checkerboard analyses with heat plots showing fractional growth at various combinations of BAP and D-Ser; **b)** Isobologram analyses (see Fig S8 for explanation); light gray region indicates additivity (FIC_I > 0.5 and < 1.0), blue region indicates synergy ($FIC_I \leq 0.5$), and white region indicates no relationship. FIC_I were observed in the range 0.32 – 1.03 across all experiments for all concentrations.

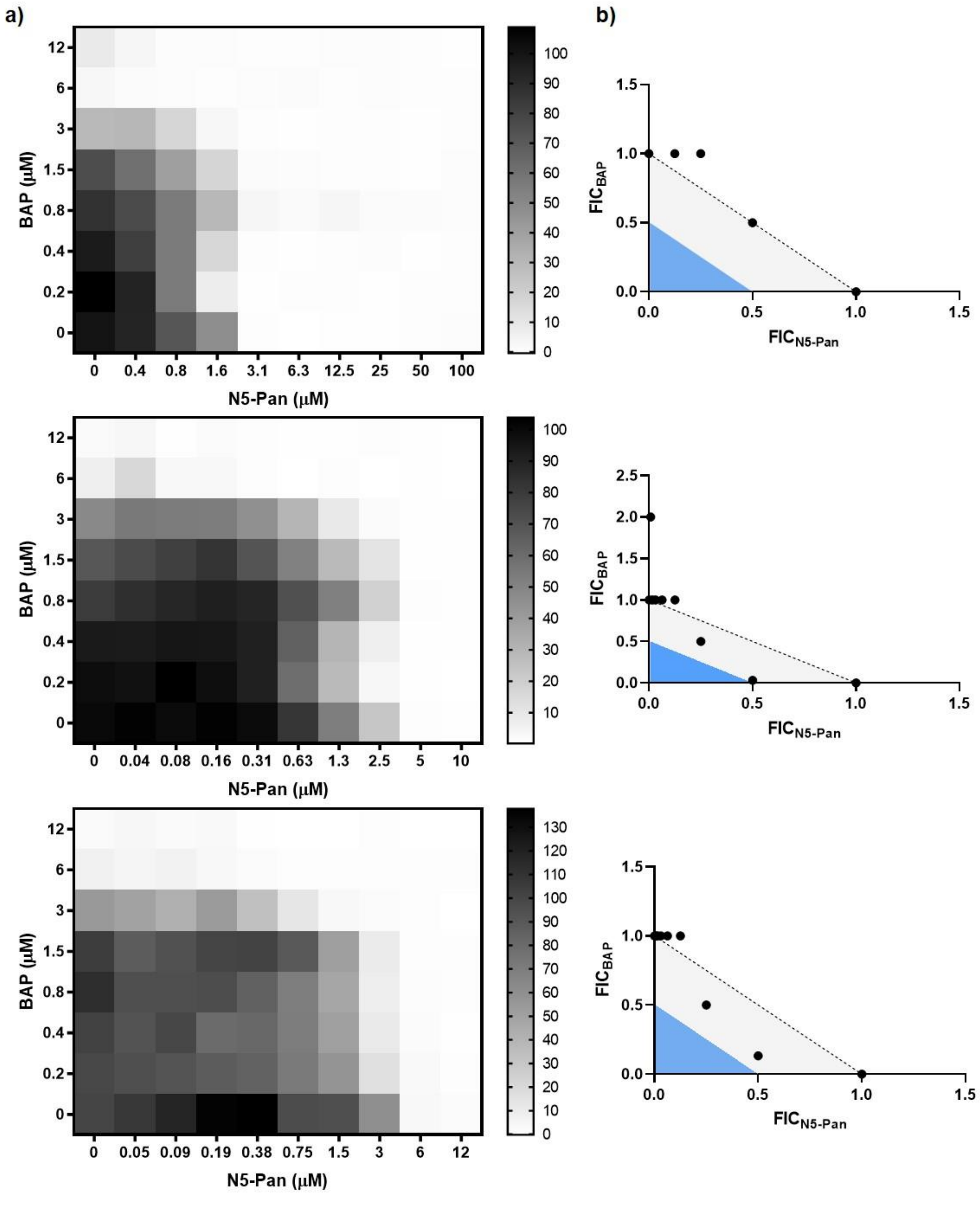

Figure S5. Replicate checkerboard and isobologram analyses for the BAP/N5-Pan combination studies in MOPS-glycerol show an additive-to-indifferent relationship (see Fig 6a). **a)** Replicates of checkerboard analysis showing heat plots with fractional growth at various combinations of BAP and N5-Pan; **b)** Corresponding replicate isobolograms (see Fig S8 for explanation); light gray region indicates additivity (FIC_I > 0.5 and < 1.0), blue region indicates synergy (FIC_I ≤ 0.5). FIC_I were observed in the range 0.53 – 2.00 across experiments for all concentrations.

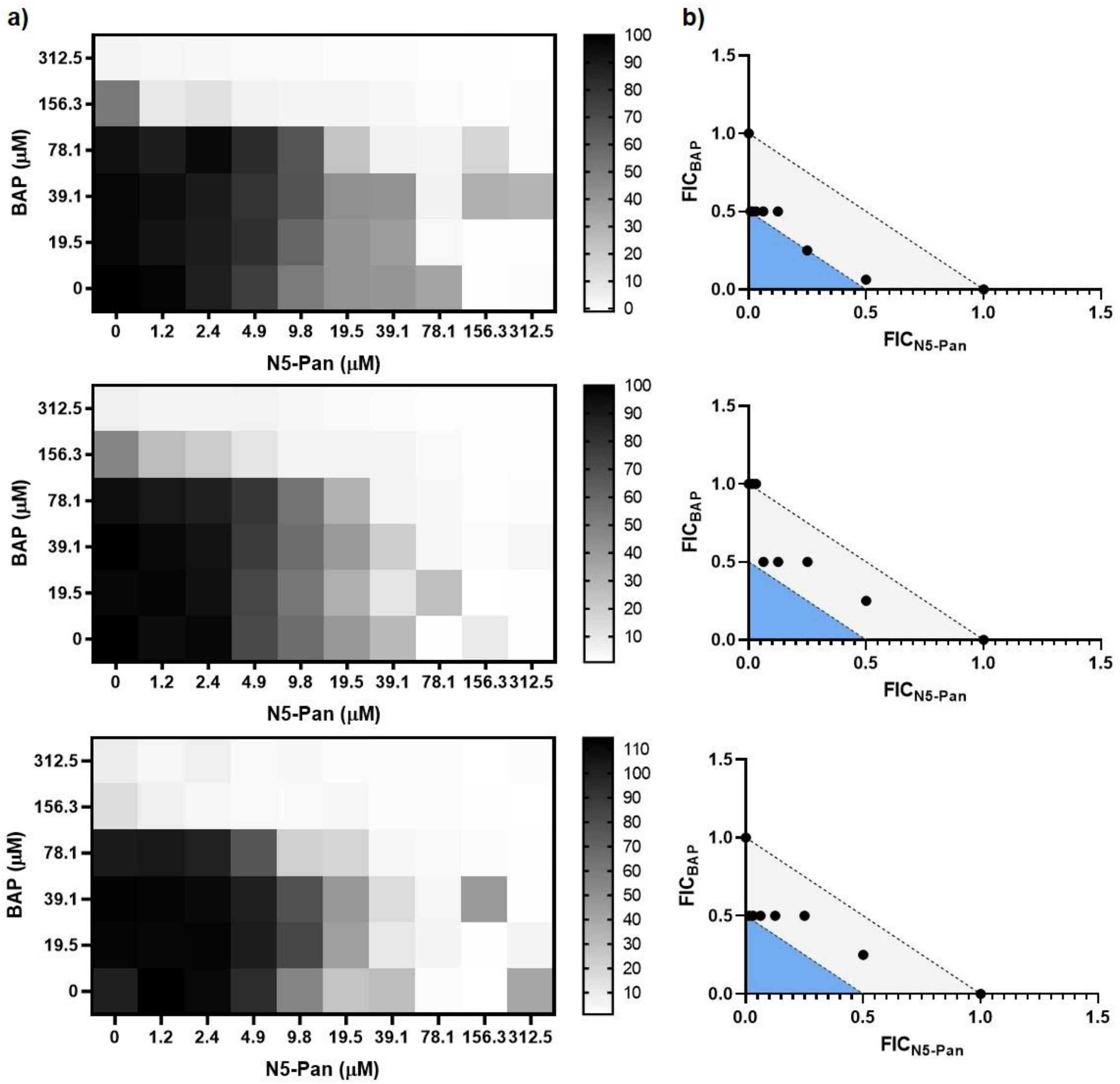

Figure S6. All replicate checkerboard and isobologram analyses for BAP/N5-Pan combination studies in M9-CasAA (see Fig 6b) show a strong additive relationship. **a)** Replicates of checkerboard analyses with heat plots showing fractional growth at various combinations of BAP and N5-Pan; **b)** Corresponding replicate isobolograms (See Fig S8 for explanation); light gray region indicates additivity (FIC_I > 0.5 and < 1.0), blue region indicates synergy ($FIC_I \leq 0.5$). FIC_I were observed in the range 0.5 – 1.03 across experiments for all concentrations.

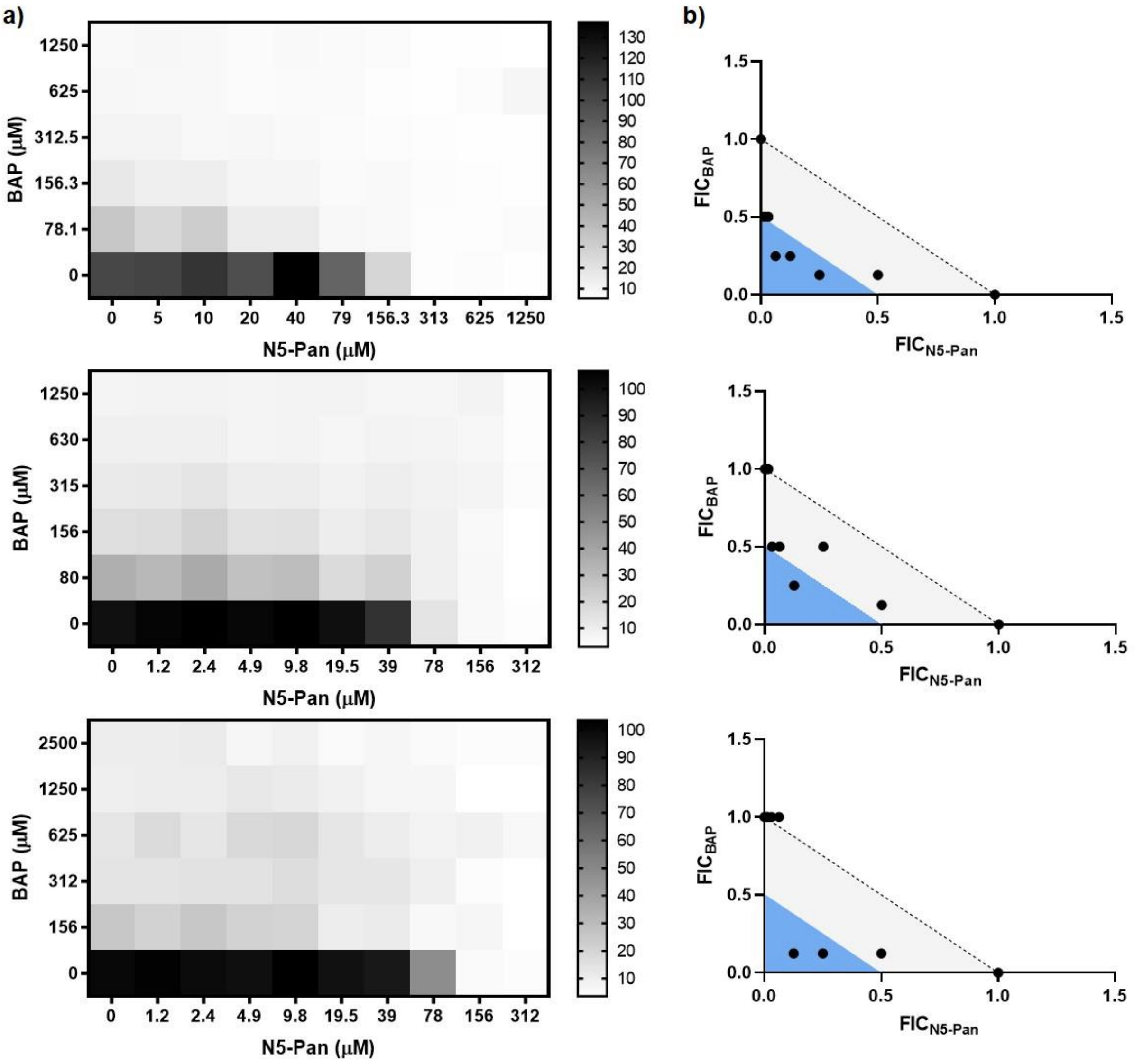

Figure S7. All replicate checkerboard and isobologram analyses for BAP/N5-Pan combination studies in pooled human urine (see Fig 6c) show an additive to synergistic relationship. **a)** Replicates of checkerboard analyses with heat plots showing fractional growth at various combinations of BAP and N5-Pan; **b)** Corresponding replicate isobolograms (See Fig S8 for explanation); light gray region indicates additivity (FIC_I > 0.5 and < 1.0), blue region indicates synergy ($FIC_I \leq 0.5$). FIC_I were observed in the range 0.25 – 1.01 across experiments for all concentrations.

$$
\begin{aligned}
\text{FIC index (FIC}_I) &= \text{FIC}_A + \text{FIC}_B \\
&= ([A]/\text{MIC}_A) + ([B]/\text{MIC}_B) \\
&= (1.6\ \mu\text{M}/12.5\ \mu\text{M}) + (6\ \mu\text{M}/12\ \mu\text{M}) \\
&= 0.128 + 0.5 \\
\text{FIC}_I &= 0.628
\end{aligned}
$$

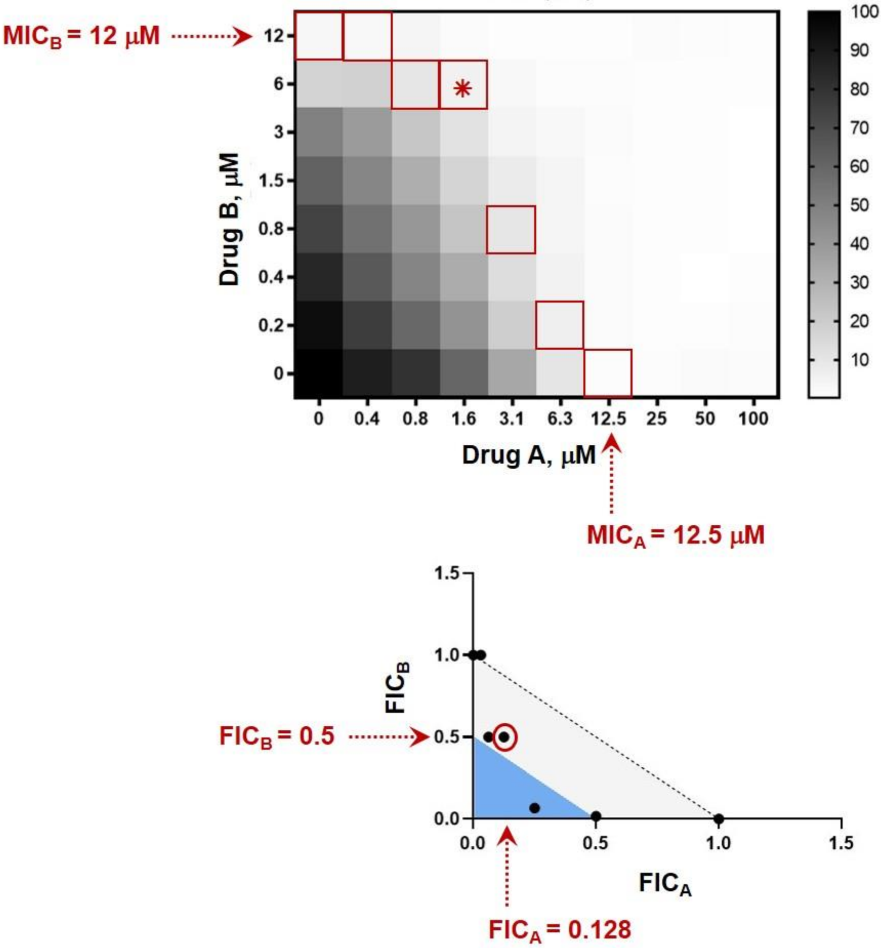

Figure S8. Method for assembling isobolograms. Red boxes indicate wells with < 10% bacterial growth compared to the (-) drug control, over the range of Drug A concentrations tested in combination with Drug B. Calculation of FIC_A, FIC_B and FIC_I is shown for a selected drug combination (✳). This combination of Drug A & Drug B is represented by a single point on the isobologram (circled in red), where $FIC_A = 0.128$ and $FIC_B = 0.5$. This analysis was repeated for each growth inhibitory drug combination of the checkerboard analysis (wells boxed in red), to generate an isobologram. The FIC_I for this particular combination of Drug A and Drug B is 0.628 (light grey region of the isobologram, additive). The FIC_I range for a given checkerboard analysis was determined by calculating FIC_I for each combination notated by the red boxes.

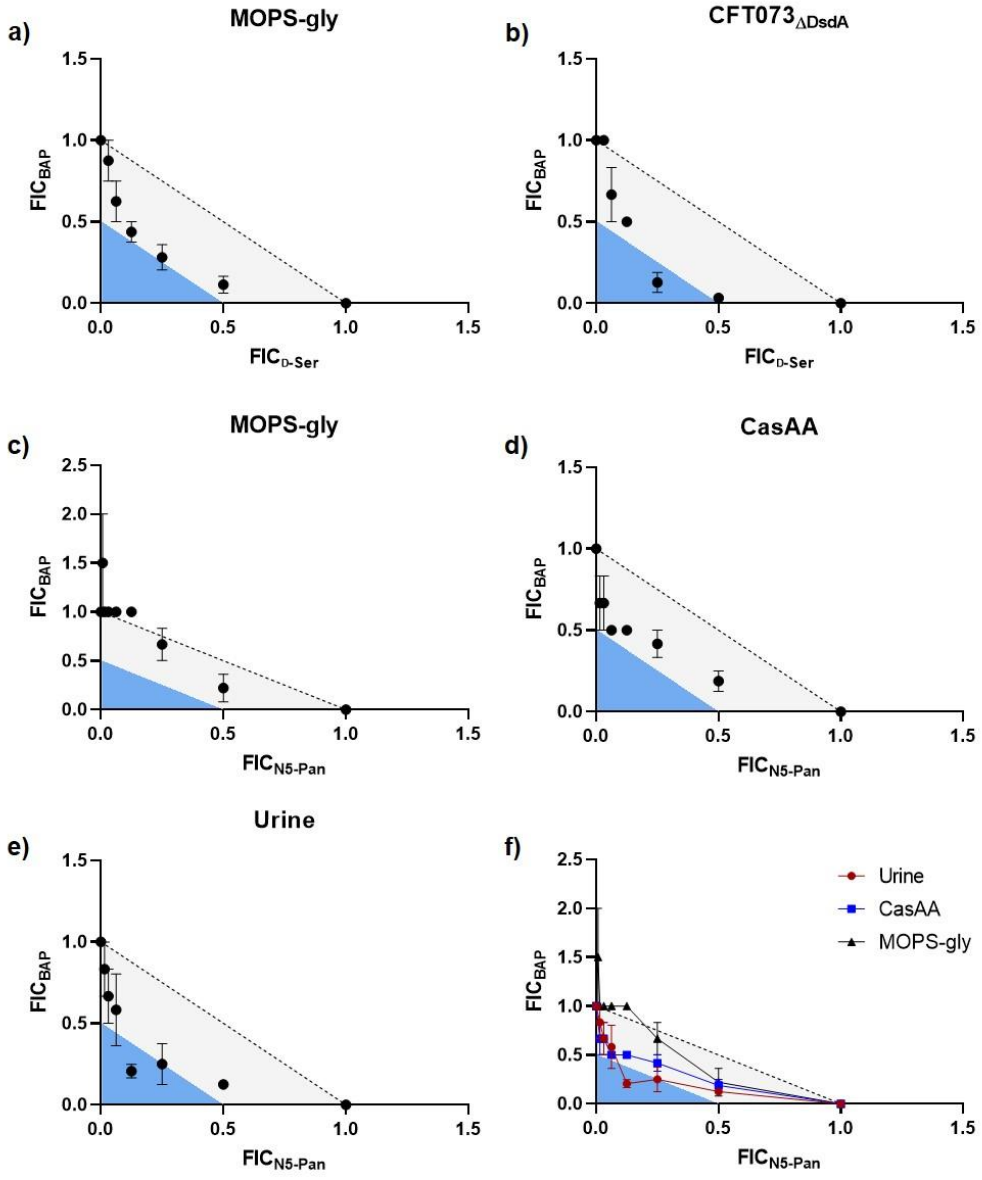

Figure S9. Isobolograms with averaged FIC values, depicting inhibitor relationships under different conditions. **a)** Isobologram with averaged FIC values showing an additive-to-synergistic relationship of the BAP/d-Ser combination in MOPS-glycerol ($p < 0.0001$); **b)** Isobologram with averaged FIC values showing an additive-to-synergistic relationship of the BAP/d-Ser combination against CFT073$_{\Delta DsdA}$ ($p = 0.0028$); **c)** Isobologram with averaged FIC values showing an additive-to-indifferent relationship of the BAP/N5-Pan combination in MOPS-glycerol ($P = 0.455$); **d)** Isobologram with averaged FIC values showing a strong additive effect of combining BAP and N5-Pan in CasAA ($p < 0.0001$); **e)** Isobologram with averaged FIC values showing an additive-to-synergistic relationship of the BAP/N5-Pan combination in urine ($p = 0.0003$); **f)** Combined isobologram analysis showing how the BAP/N5-Pan relationships are different in MOPS-glycerol (black), CasAA (blue) and urine (red). In all cases, the statistical difference of measured FIC_I to the additive model ($FIC_I = 1$) was assessed using Wilcoxon signed rank tests.

References

1. Patrone, J.D.; Yao J.; Scott N.E.; Dotson G.D. Selective Inhibitors of Bacterial Phosphopantothenoylcysteine Synthetase. *Journal of the American Chemical Society* **2009,** *131* , 16340-16341.
2. Awuah E.; Ma E.; Hoegl A.; Vong K.; Habib E.; Auclair K.. Exploring structural motifs necessary for substrate binding in the active site of Escherichia coli pantothenate kinase. *Bioorg Med Chem* **2014,** *22*, 3083-3090.
3. Arnott, Z.L.P.; Nozaki, S.; Monteiro, D.C.F.; Morgan, H.E.; Pearson, A.R.; Niki, H.; Webb, M.E. The Mechanism of Regulation of Pantothenate Biosynthesis by the PanD-PanZ·AcCoA Complex Reveals an Additional Mode of Action for the Antimetabolite N-Pentyl Pantothenamide (N5-Pan). *Biochemistry* **2017**, *56*, 4931–4939.
